# Factors associated with declining a menstrual cup among female students and their parents in Ugandan secondary schools: a cross-sectional study

**DOI:** 10.1136/bmjopen-2024-087438

**Published:** 2024-12-05

**Authors:** Levicatus Mugenyi, Mandikudza Tembo, Kate Andrews Nelson, Katherine A Thomas, Catherine Kansiime, Stephen Lagony, Alex Muleyi Mpaata, Sophie Belfield, Shamirah Nakalema, Agnes Akech, Belen Torondel-Lopez, Helen A Weiss

**Affiliations:** 1 Statistics, MRC/UVRI and LSHTM Uganda Research Unit, Entebbe, Uganda; 2 London School of Hygiene & Tropical Medicine, London, UK; 3 Biomedical Research and Training Institute, Harare, Zimbabwe; 4 MRC/UVRI and LSHTM Uganda Research Unit, Entebbe, Wakiso, Uganda; 5 WoMena Uganda, Kampala, Uganda; 6 London School of Hygiene and Tropical Medicine, London, UK; 7 Epidemiology and Population Health, London School of Hygiene and Tropical Medicine, London, London, UK; 8 London School of Hygiene and Tropical Medicine

**Keywords:** Menstrual health, menstrual products, menstrual cup, adolescents, Sub-Saharan Africa

## Abstract

**Introduction:**

A greater choice of menstrual products may improve menstrual health (MH). This study assessed factors associated with declining consent to receive a menstrual cup by parents and female students participating in a MH intervention trial in Ugandan schools.

**Methods:**

We analysed baseline data from a cluster-randomised trial evaluating the effectiveness of a multicomponent MH intervention among female students in 60 Ugandan secondary schools. Parental consent and student assent to receive a menstrual cup and training on its use was sought separately from consent from other trial activities. Random-effects logistic regression models were used to estimate adjusted OR (aOR) and 95% CIs for factors associated with (i) parents or guardians declining the cup and (ii) students declining the cup using hierarchical conceptual frameworks.

**Results:**

The baseline trial population comprised 3705 post-menarchal students (mean age 15.6 (SD 0.9 years), of whom 2048 (55.3%) were day students. Among the parents of the 3635 participants aged <18 years, 1566 (43.1%) declined consent for their student to receive the cup. This was higher in Wakiso District than in Kalungu District (52.9% vs 8.0%, p<0.001). Parental decline of the cup differed by ethnicity, and this association varied between districts (p=0.004). Overall, 20.5% students declined the cup (Kalungu 21.1%, Wakiso 20.2%, p=0.62). Student decline of the cup was higher among day than boarding students (aOR=1.40, 95% CI 1.07 to 1.84), those with academic performance above the median score (aOR=1.29, 95% CI 1.01 to 1.65), those whose menstrual practice needs score was above the median (aOR=1.36, 95% CI 1.08 to 1.72) and those with more negative attitudes to MH (aOR=1.46, 95% CI 1.16 to 1.83).

**Conclusion:**

Among Ugandan students and their parents, declining consent to receive a menstrual cup varied by district and ethnicity as well as academic performance and menstrual-related factors. A contextual understanding of the barriers for uptake of the menstrual cup is needed to guide future interventions.

**Trial registration number:**

ISRCTN45461276.

**STRENGTHS AND LIMITATIONS OF THIS STUDY**

- A large population-based study with a high response rate
- Inclusion of both parental consent and student assent for the menstrual cup
- Data only on consent or assent to the cup, not usage
- No data on reasons for declining the menstrual cup
- Parental decline for the cup is indicated to be associated with ethnicity, meaning the results may not be generalisable to other settings with different sociocultural environments.

## Introduction

 Menstrual health (MH) is a human right, defined as a state of complete physical, mental and social well-being and not merely the absence of disease or infirmity in relation to the menstrual cycle.^1^ Good MH is critical to meeting the sustainable development goals (SDGs) such as gender equality (SDG5) and good health and well-being (SDG3).^2^ Many people face multiple challenges achieving good MH^3 4^ including lack of timely knowledge of menstrual practices; lack of access to safe menstrual products and effective menstrual-related pain management, inadequate water, sanitation and hygiene (WASH) facilities; and sociocultural attitudes and myths around menstruation.^5^ As a result, many women and girls experience stigma and shame, isolation, lack of confidence and embarrassment that can negatively inform school or work participation, reproductive health, social engagement and quality of life.^3 4^

Access to safe and reliable menstrual products is linked with poverty, gender inequalities and political and economic instabilities and is particularly challenging and harmful for those in low- and middle-income countries (LMICs).^3^ A systematic review found that MH interventions that facilitate access to menstrual education and products can improve MH experiences and outcomes for girls and women in LMICs.^4^ However, little is known about which factors are associated with menstrual product choice^6^—an evidence gap we address in this study.

Menstrual products may be disposable (eg, disposable pads, tampons) or reusable (eg, reusable pads, the menstrual cup). The menstrual cup is a reusable non-absorbent silicone cup that is inserted into the vagina to collect menstrual blood.^7^ It is growing in popularity in many high-income countries and more recently in some low-income settings and can last up to 10 years.^8 9^ In high-income countries, the menstrual cup has been found to be highly acceptable and more comfortable and leak-proof than other menstrual products.^8 10^ There are relatively few studies on menstrual cup use in LMICs. One study, on sexual reproductive health services and menstrual product choice, offered 27 725 young women aged 16–24 years in Zimbabwe, the choice between either a menstrual cup or a four-pack of reusable pads.^11^ Of the 25 433 women who chose a menstrual product, only 7.2% chose the cup.^11^ Most participants feared that insertion of the cup may be painful or might perforate the hymen, thus affecting their virginity.^11^ In a qualitative study among 141 women and girls in Malawi, participants found the menstrual cup is complicated to use; however, due to the long-term affordability and longevity of them, they were still an appealing option.^9^ Another quantitative study among 509 further education students aged 18–24 years in South Africa found that about half (50.5%) students reported cups to be uncomfortable and difficult to insert; however, most students reported that they would continue to use the cup at each follow-up visit.^7^ In a study in a rural area of Western Kenya among 192 girls aged 14–16 years provided with cups, 70.9% of girls had evidence of using the cup (assessed by change in colour of the cup) with a median follow-up time of 9 months.^12^ In Uganda, a pilot study (MENISCUS-2) of a MH intervention was not able to include menstrual cups due to concerns by the national ethics committee that the cup could lead to damage of the hymen and effect a girl’s virginity.^13^

Reusable menstrual cups may be a sustainable and long-term cost-effective menstrual product choice in LMICs, but there is little evidence on how parental and individual MH-related factors affect willingness to receive the cup in this context.^7 8^ The aim of this study was to investigate the sociodemographic characteristics of parents and MH-related characteristics of girls associated with providing consent/assent for girls to receive a menstrual cup, as part of a multicomponent MH intervention in Ugandan secondary schools. This will help us understand barriers and facilitators of menstrual cup use, which is of public health importance as cups are being increasingly offered to people who menstruate.^12^ We will report in detail on MH in this population, including the impact of the intervention on MH, in subsequent papers.

## Methods

### Study design and setting

This study uses baseline data from a cluster-randomised trial evaluating the effect of a multicomponent MH intervention (‘MENISCUS’) on education, health and well-being among secondary school girls in Uganda.^14^ Baseline data were used to assess factors associated with declining consent to receive a menstrual cup by parents and female students participating in the trial. The intervention comprised: (i) training teachers to improve puberty education; (ii) a student-led drama skit to reduce stigma and improve knowledge of MH; (iii) training school members to deliver menstrual-health education sessions alongside the distribution of a menstrual kit containing reusable menstrual pads and the optional menstrual cup; (iv) training on pain management, including provision of analgesics; and (v) improvements to school water and sanitation (WASH) facilities. In each school, a ‘Menstrual Health Action Group’, consisting of teachers, students and/or parents, was established to help coordinate and sustain the intervention. Details of the trial design and procedures have been published previously.^14^

The trial was conducted in Wakiso and Kalungu districts in Uganda. Wakiso district that partly encircles Kampala, the Uganda’s capital city and Kalungu district is approximately 130 kilometers in the southwest of Kampala and is largely a rural setting. Parental consent was conducted between November 2021 and July 2022 and student assent/consent and baseline data collection between March and July 2022. The trial included opt-in consent/assent for a menstrual cup receival and training, separate to consent for the main trial procedures. This enables us to assess characteristics of female students who did not consent/assent to cup use.

### Study population

The trial took place in 60 randomly selected secondary schools (44 in Wakiso district and 16 in Kalungu district). Inclusion criteria for the schools were: mixed-sex secondary schools with students in secondary one (S1)–S4; day or mixed day/boarding schools; at least minimal WASH facilities (definition aligned with Joint Monitoring Programme which includes an improved water source and sex-specific sanitation facilities that are functional, usable and accessible to female students);^15^ and estimated enrolment of approximately 50–125 female S1 students in Wakiso and 40–125 female S1 students in Kalungu.^14^ Exclusion criteria were schools that were currently participating in a MH-related programme; boarding schools with no day students; single-sex schools; and schools exclusively for students with disabilities.^14^

All female students in S2 in 2022 who were present at the time of the baseline survey, whose parent/guardian provided consent (if aged <18 years) and who assented/consented to the trial and intervention procedures (with or without the cup) were included in the trial. In this paper, only female students who had started menstruating were included.

### Participant consent and assent

Prior to randomisation, trained research team members sought (i) written informed school-level consent from head teachers or directors to cover school-level research and intervention activities and (ii) written informed parent/guardian consent for eligible female students aged under 18 years through information meetings held at each school. Parents/guardians who were unable to attend information meetings at school were reached through phone calls to seek documented verbal informed opt-in consent. Research team members seeking consent and assent were trained in the use of the menstrual cup to respond to participants’ concerns and questions.

Trained research assistants then asked female students with parental consent (or those aged 18 or older) to watch information videos explaining trial procedures on a tablet with headphones, with a choice of English or Luganda (the local language), and to then answer questions to assess their understanding of the trial procedures. If the student understood the video and agreed to participate, they were asked to provide electronic assent by signing a form on the tablet. Participants were given a paper copy of the assent form and information sheet to retain.

For both parents and female students, a separate opt-in informed consent/assent form was administered for provision of the menstrual cup and associated training, so parents and students could opt to participate in all trial activities except for receiving a cup. The separate consent for the cup was due to our earlier formative research in Wakiso District which indicated that the menstrual cup may not be acceptable to many parents.^13^

### Public involvement

The public was involved in the research design before the trial started through stakeholder workshops (August 2016 and October 2018) and participating in a Theory of Change workshop in April 2017. The stakeholders included teachers, students, parents and representatives from the Ministry of Education and Sports (MoES), Ministry of Health (MoH), the District Education Officials, Makerere University and Non-Government Organisations working on MH. Discussions from these workshops informed modifications in the trial design and intervention. Stakeholders were involved in the recruitment and conduct of the study through discussions with District Education Officials and information sessions for headteachers and parents. The results from this study will be disseminated to all stakeholders including students, teachers, parents, Districts Education Officials, and policymakers at the MoH and MoES through workshops.

### Baseline data collection

Trial participants were asked to complete a self-administered questionnaire on tablets using Open Data Kit (ODK) software. Data included sociodemographic characteristics, caregiver characteristics, household-level characteristics, as well as female students’ knowledge of and attitudes about puberty and menstruation and their experiences with menstrual periods measured by the Menstrual Practice Needs Scale (MPNS) score and the Self-Efficacy in Addressing Menstrual Needs Scale (SAMNS-26) score.^16 17^ The 36-item MPNS tool is a comprehensive measure of menstrual self-care experience including access to sufficient, comfortable materials to catch or absorb bleeding, supportive spaces for managing menstruation and for disposal and laundering of used materials.^16^ The 26-item SAMNS score measures adolescents’ confidence in their capabilities to address their menstrual needs.^17^

Data were collected from all female students who consented to the trial (including those who did not consent to the cup), meaning that data on characteristics of those who declined to receive the cup were available for analysis. The ethnicity and religion of the primary caregiver were assumed to be the same as for the corresponding student. However, data on caregiver’s relationship with the student were obtained from the caregiver that provided parental consent, who may not be the primary caregiver.

### Data management

Each day during data collection, the research assistants checked data for completeness and synced it to the ODK Central Server database. The trial statistician exported the data to Stata V.18 software for further cleaning and analysis.

### Outcome measures

For this paper, the outcomes of declining the menstrual cup among parents and students respectively were defined by (1) the proportion of female students aged under 18 years whose parents or guardians declined consent for their daughter to receive the cup, among those who consented to the trial (‘parental decline’) and (2) the proportion of female students who declined assent/consent to the cup, among those whose parents had consented to the cup or who were aged 18 years and over (‘student decline’).

### Statistical analyses

Analyses were done using Stata V.18 software and R V.4.2.3. Baseline characteristics of study participants were summarised by district using proportions for categorical data and means (SD) or medians (IQR) for continuous data. Socioeconomic status (SES) was derived using principal component analysis (PCA), with the first quintile representing the highest SES and the fifth quintile the lowest SES.^18^ The variables considered in the PCA included ownership of a computer, household furniture, more than 10 chickens or birds, animal (eg, cows, goats, sheep, pigs), moving motorbike, moving car, land for farming, access to electricity, type of toilet and main source of water.

The MPNS score on menstrual experience during the last menstrual period was calculated as the average of (i) the core items and school-specific items and (ii) the relevant material-specific items, since participants answer a different set of items depending on the type of materials reported.^16^ For each item, participants were asked how often the statement was true during their last menstrual period (always, often, sometimes, never). Negatively phrased items were reverse coded so that a higher MPNS score corresponded to fewer unmet menstrual needs. The median MPNS score was used as a cut-off to categorise participants as having low or high scores, corresponding to many or few unmet menstrual needs, respectively. Single imputation using school-level means was used to impute the 1% missing data on MPNS score.

The SAMNS-26 score on menstrual confidence was calculated as the mean of the 26 items.^17^ Each item was on a scale of 0–10 with 0 representing the least self-efficacy. Participants were categorised as having high or low self-efficacy by whether their SAMNS-26 score was above or below the median score.

Mental health problems were assessed by the Strength and Difficulties Questionnaire (SDQ) Total Difficulties score.^19^ The SDQ is widely used to assess mental health problems for children and adolescents.^19^ The minimum and maximum scores are 0 and 40, respectively, with higher values representing increasing mental health problems. As there are no validated Ugandan cut-offs for the SDQ score,^20^ we used the median cut-off for analyses in this paper.

Academic performance was assessed by the Uganda National Examination Board (UNEB) using mathematics and biology questions covering material taught in all schools in S1 and first term of S2. A standardised normal score (z-score) was generated for each subject, and the mean z-scores for both subjects combined was calculated to obtain the UNEB assessment score. Students were categorised as having low or high academic achievement if their UNEB assessment score was below or above the median. School-level means were used to impute the 11% missing data on UNEB assessment scores.

Other factors considered to be potentially associated with declining the menstrual cup were sociodemographic characteristics of parents/guardians and students, food insecurity, being a day versus boarding student, knowledge and attitudes about menstruation, age at first menstruation, knowledge of menstruation before menarche and school-level factors (availability of WASH facilities at school (eg, water in a toilet block, separate toilet blocks for female and male students, and a private menstrual changing space) and school ownership (government or private).

**Figure 1:**
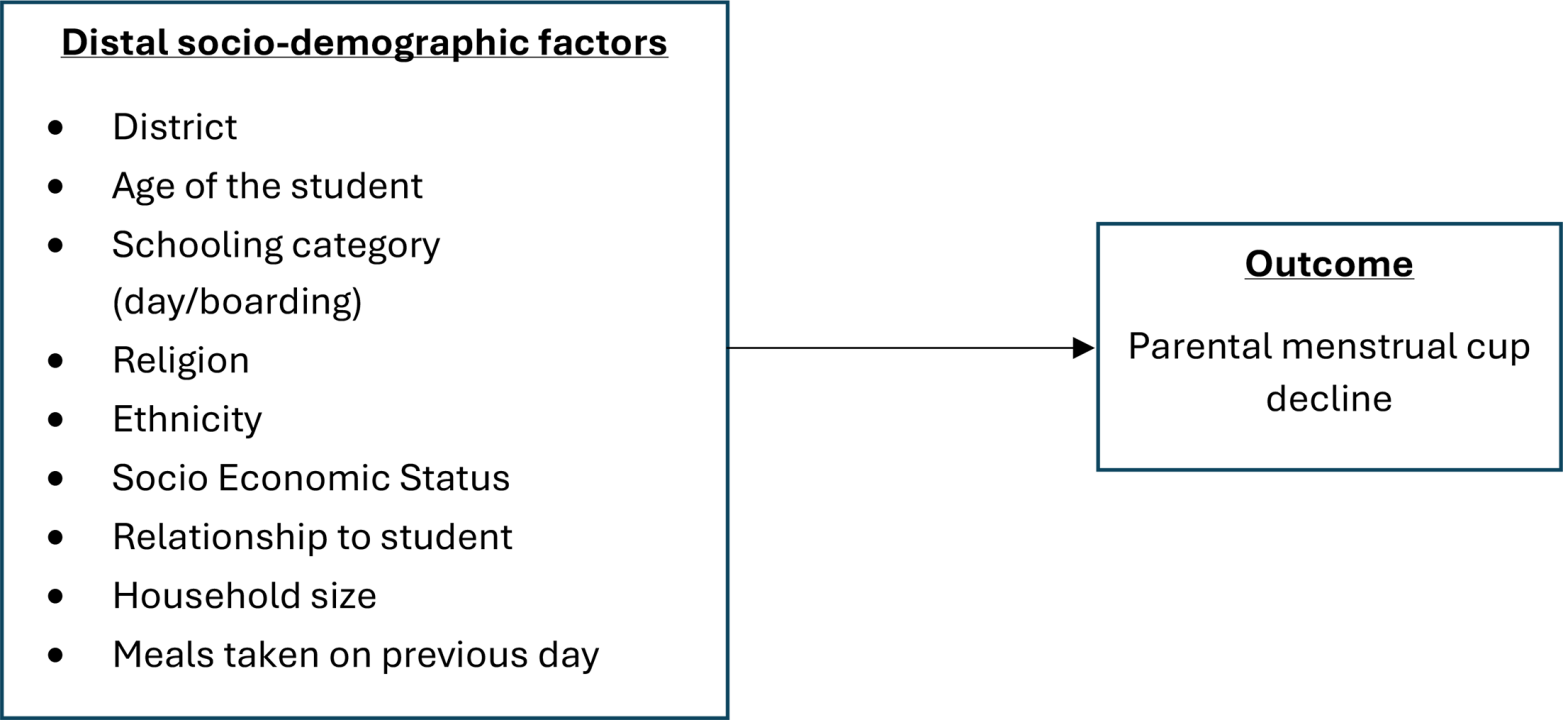
Conceptual framework showing plausible relationships between family-level factors and the parental-level cup decline.

**Figure 2:**
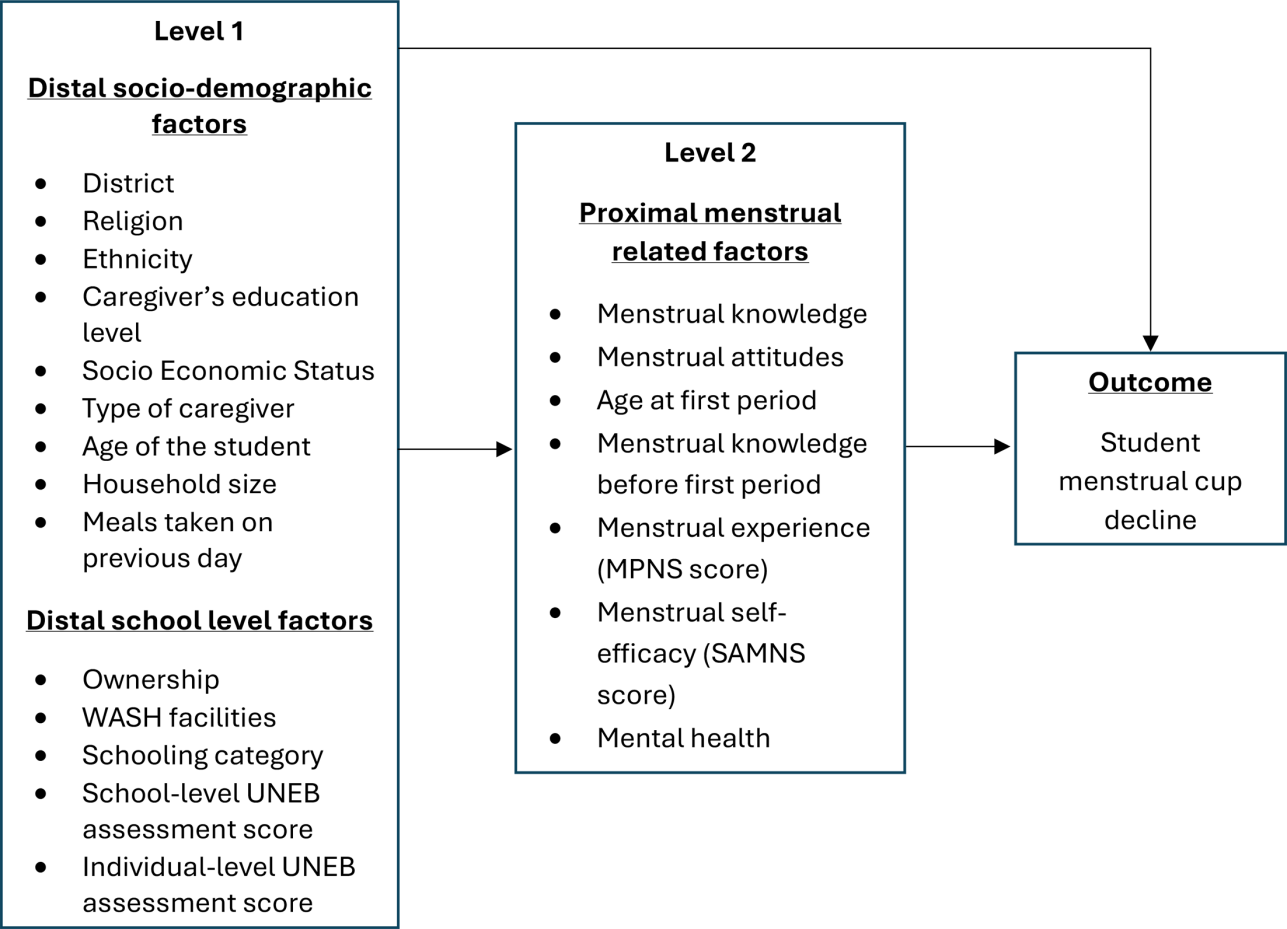
Conceptual framework showing plausible relationships between female student-level factors and the student-level cup decline, among those whose parents consented to the cup.

We used hierarchical conceptual frameworks to guide our models,^21^ including exposure variables found in the literature to be associated with cup use (ie, caregiver education, economic status, knowledge of puberty and menstruation and experiences related to menstruation).^3^ Figure 1 shows the factors that may affect parental menstrual cup decline, and figure 2 shows the factors that may affect student menstrual cup decline (among those whose parents have consented or were aged >18). We assume that distal sociodemographic and school-level factors (level 1) may influence more proximal factors (eg, knowledge and attitudes towards menstruation, menstrual practices, self-efficacy and mental health problems) (level 2), which may in turn affect female student menstrual cup decline.

We used random-effects logistic regression to estimate ORs and 95% CIs for the associations between cup decline and exposure variables, adjusted for school-level clustering. We identified district a priori as a potential effect-modifier. We used univariable analyses to assess associations of exposure variables in the conceptual frameworks (3) with the outcomes using the likelihood ratio test (LRT) to assess strength of evidence.

To build the multivariable models for parental and student cup decline, respectively, sociodemographic factors for the parental outcome (figure 1) and sociodemographic and school-level factors for the student outcome (level 1; figure 2) were included in the respective models for each outcome. Variables in level 1 were adjusted for each other and were retained as a core group of level 1 factors if independently associated with the outcome (p<0.1). For the student outcome, level 2 factors were then included in the model adjusted for each other, and for the core group of level 1 factors. They were retained in the final model if independently associated with the outcome (p<0.1).

We used the final models to estimate the intracluster correlation (ICC) in menstrual cup decline among parents of female students within the same school and among female students within the same school.

## Results

A total of 4281 female students were eligible for the trial, of whom 3841 (89.7%) were enrolled. Of those not enrolled (n=399), more than half (n=238, 59.6%) of their parents did not consent to the trial, 149 (37.3%) were not present at baseline, and 12 (3.0%) students did not assent/consent to the trial themselves. Of the enrolled students, 3705 (96.5%) had started menstruating and were included in this study.

A total of 3635 (98.1%) enrolled students were aged <18 years (and had parental consent for the trial), and the remaining 70 (1.9%) were >18 years and provided their own consent for the trial (table 1). The mean age was 15.6 (SD=0.9) years, with approximately half the students (55.3%) being day students, and the majority (68.9%) from the Muganda ethnic group. The mean household size was 7.1 (SD=3.1); 17.9% experienced food insecurity (ie, reported to have had one or no meals the previous day). SES varied by district with 26.6% of participants in Kalungu versus 18.1% of those in Wakiso being in the lowest SES quintile (table 1).

**Table 1:** Baseline socio-demographic characteristics of female study participants by district

	Kalungu	Wakiso	Total
Number of participants	822 (22.2%)	2883 (77.8%)	3705 (100.0%)
Parental consent for trial			
Yes obtained (aged<18 years) *	797 (97.0%)	2838 (98.4%)	3635 (98.1%)
Not required (aged 18+)	25 (3.0%)	45 (1.6%)	70 (1.9%)
Age in years, mean (SD)	15.7 (0.9)	15.6 (0.9)	15.6 (0.9)
Age category (years)			
<15	49 (6.0%)	284 (9.9%)	333 (9.0%)
15	287 (34.9%)	1210 (42.0%)	1497 (40.4%)
16	342 (41.6%)	1022 (35.4%)	1364 (36.8%)
17	116 (14.1%)	275 (9.5%)	391 (10.6%)
18+	28 (3.4%)	92 (3.2%)	120 (3.2%)
Schooling category			
Day	381 (46.4%)	1667 (57.8%)	2048 (55.3%)
Boarding	441 (53.6%)	1216 (42.2%)	1657 (44.7%)
Religion			
Catholic	383 (46.6%)	793 (27.5%)	1176 (31.7%)
Protestant/Born Again/SDA	241 (29.3%)	1205 (41.8%)	1446 (39.0%)
Muslim	195 (23.7%)	871 (30.2%)	1066 (28.8%)
None/other	3 (0.4%)	14 (0.5%)	17 (0.5%)
Ethnicity			
Muganda	610 (74.2%)	1942 (67.4%)	2552 (68.9%)
Non-Muganda	212 (25.8%)	941 (32.6%)	1153 (31.1%)
Household size, mean (SD)	7.4 (3.9)	7.0 (2.9)	7.1 (3.1)
Number of meals eaten on the previous day			
One or fewer	143 (17.4%)	520 (18.0%)	663 (17.9%)
Two	441 (53.6%)	1440 (49.9%)	1881 (50.8%)
Three or more	238 (29.0%)	923 (32.0%)	1161 (31.3%)
Social economic status (SES)†			
Lowest	219 (26.6%)	522 (18.1%)	741 (20.0%)
Medium-low	210 (25.5%)	545 (18.9%)	755 (20.4%)
Medium	159 (19.3%)	572 (19.8%)	731 (19.7%)
Medium-high	147 (17.9%)	592 (20.5%)	739 (19.9%)
Highest	87 (10.6%)	652 (22.6%)	739 (19.9%)

* Includes 19 female students aged 18 years and above who requested their parents’ consent first.

† Includes ownership of assets (like computer, furniture, land, etc.),; animals and chicken/birds; availability of electricity or solar; source of water; and type of toilet.

SDASeventh-day Adventist

Parental consent and student assent for the menstrual cup are shown in online supplemental figure 1. Overall, 43.1% (1566/3635) female students had parents who declined consent for the menstrual cup, and this proportion was substantially higher in Wakiso (52.9%) than in Kalungu (8.0%) (p<0.001).

Of the 2069 female students whose parents consented to the menstrual cup, 20.6% (n=426) declined assent for the cup. This was similar by district (Kalungu, 21.3% (156/733); Wakiso, 20.2% (270/1336); p=0.57). Of the 70 female students aged >18 years who did not require parental consent, 18.6% (n=13) declined consent for the cup. Combining students whose parents provided consent and those who did not require parental consent, 20.5% (439/2139) students declined consent/assent for the menstrual cup, and this was similar by district (Kalungu, 21.1% (160/758); Wakiso: 20.2% (279/1381); p=0.62).

### Parental cup decline and associated factors

The proportion of parents declining the cup was similar by type of parent/guardian (mother, 43.2%; father, 39.2%; guardian, 45.5%; p=0.59). In Wakiso, there was strong evidence that the proportion of parents/guardians who declined was lower among the non-Muganda than the Muganda ethnic group (46.3% vs 56.1%; adjusted OR (aOR)=0.69, 95% CI 0.58 to 0.82, p<0.001). Conversely in Kalungu, the proportion who declined was higher among the non-Muganda than the Muganda (11.1% vs 6.9%; aOR=1.66, 95% CI 0.95 to 2.88, p=0.07). There was strong evidence that the association of ethnic group with parental decline varied by district (p=0.004). There was little evidence that parental decline was associated with age of the student (p=0.09), and no evidence of an association with other sociodemographic or socioeconomic factors (table 2).

**Table 2:** Factors associated with parental menstrual cup decline

	TotalN (%)	Declined cupN (%)	UnadjustedOR (95% CI)	LRT p value	Adjusted*OR (95% CI)	LRT p value
N	3635	1566 (43.1)				
Relationship†						
Mother	1958 (53.9)	848 (43.3)	1.00			
Father	694 (19.1)	272 (39.2)	0.90 (0.73, 1.10)	0.59		
Guardian	981 (27.0)	446 (45.5)	0.96 (0.80, 1.16)			
District/ethnicity						
Kalungu	(n=797)					
Muganda	591 (74.1)	41 (6.9)	1.00		1.00	
Non-Muganda	206 (25.9)	23 (11.1)	1.67 (0.96, 2.88)	0.07	1.66 (0.95, 2.88)	0.07
Wakiso	(n=2838)					
Muganda	1916 (67.5)	1075 (56.1)	1.00		1.00	
Non-Muganda	922 (32.5)	427 (46.3)	0.69 (0.58, 0.82)	<0.001	0.69 (0.58, 0.82)	<0.001
Student age in years						
≤15	1830 (49.4)	804 (43.9)	1.00			
>15	1875 (50.6)	762 (42.2)	0.99 (0.85, 1.17)	0.94		
Schooling category						
Day	1997 (54.9)	866 (43.4)	1.00			
Boarding	1638 (45.1)	700 (42.7)	1.10 (0.92, 1.33)	0.30		
Religion						
Catholic	1145 (31.5)	457 (39.9)	1.00			
Protestant/born again/SDA	1418 (39.0)	639 (45.1)	0.89 (0.74, 1.08)	0.66		
Muslim	1055 (29.0)	462 (43.8)	0.98 (0.77, 1.23)			
None/other	17 (0.5)	8 (47.1)	0.83 (0.26, 2.61)			
Social economic status (SES)						
Lowest	719 (19.8)	305 (42.4)	1.00			
Medium-low	738 (20.3)	284 (38.5)	0.80 (0.62, 1.03)	0.43		
Medium	717 (19.7)	313 (43.7)	0.89 (0.69, 1.14)			
Medium-high	728 (20.0)	325 (44.6)	0.95 (0.74, 1.23)			
Highest	733 (20.2)	339 (46.3)	0.97 (0.75, 1.25)			
Household size						
<5 people	1114 (30.6)	492 (44.2)	1.00			
6–10 people	2179 (59.9)	924 (42.4)	0.99 (0.83, 1.17)	0.90		
10+people	342 (9.4)	150 (43.9)	1.05 (0.79, 1.40)			
Number of meals eaten on the previous day						
One or fewer	649 (17.9)	277 (42.7)	1.00			
Two	1844 (50.7)	804 (43.6)	0.96 (0.78, 1.19)	0.81		
Three or more	1142 (31.4)	485 (42.5)	0.93 (0.74, 1.17)			

* Adjusted for district, ethnicity and their interaction.

† Relationship of person consenting for the student (relationship data was missing for 2two students).

LRTlikelihood ratio testSDASeventh-day Adventist

### Student outcome and associated factors

In multivariable analyses, cup decline by students was independently associated with poorer MH attitude scores (22.7% vs 18.3% for those with <2 vs >2 positive responses (out of 3); aOR=1.46, 95% CI 1.16 to 1.83, p=0.001), being a day student (20.8% vs 20.2% for day vs boarding; aOR=1.40, 95% CI 1.07 to 1.84, p=0.01), having a higher UNEB examination score (22.7% vs 18.3% for high vs low score; aOR=1.29, 95% CI 1.01 to 1.65, p=0.04), having a high MPNS score (23.0% vs 18.4% for high vs low score; aOR=1.36, 95% CI 1.08 to 1.72, p=0.01) and not knowing their caregiver’s education (25.7% vs 18.7% for those who reported caregivers had primary or less education; aOR=1.65, 95% CI 1.16 to 2.35) (online supplemental table 1).

### Intracluster correlation

Conditional on fixed-effects covariates, we estimated correlation in menstrual cup decline as ICC=0.18 (95% CI 0.12 to 0.26) among parents of female students within the same school and as ICC=0.22 (95% CI 0.14 to 0.32) among female students within the same school,

## Discussion

This paper addresses a gap in the literature on characteristics associated with declining consent for a menstrual cup in a Sub-Saharan African setting, among parents and school-going female adolescents. To our knowledge, this is the first study to assess factors associated with parental and female student consent/assent for the cup and the first to our knowledge to assess the associations of cup consent with multiple dimensions of MH using validated measures. In a context where negative views about menstruation and menstrual practices are pervasive, understanding parental views as well as those of the students are essential to understand the context of attitudes towards menstrual product choice. Our results indicate a large difference in declining the cup by parents between districts, and effect-modification between district and ethnicity, with those of non-Muganda ethnicity being less likely to decline the cup in Wakiso than those of Muganda ethnicity, and vice versa in Kalungu. Student cup decline was greater among day scholars, those with above-average academic performance, above-average MPNS scores (ie, fewer unmet menstrual practice needs), poorer menstrual attitude scores and those who did not know the education level of their caregivers.

Our results support previous research highlighting the role of sociocultural norms in influencing menstrual product choice. For example, a study in Zimbabwe indicated that sociocultural factors informed product choice among females aged 16–24 years and that barriers to cup uptake included fears around the difficulties of insertion and concerns that the cup would compromise virginity.^6^ The difference in menstrual cup decline among parents with Muganda and non-Muganda ethnicities, respectively, could be because of cultural differences and beliefs, such as the protection of virginity among young girls, which is common for Muganda ethnic group.^22^ Kalungu residents are predominantly from the indigenous Muganda ethnic group, and Wakiso is a mixture of ethnic groups since it encircles Kampala, the capital city of Uganda. The difference by district is also likely to be due to trust in the Medical Research Council (MRC), who have worked with the local community, conducting medical research and providing medical services in rural Kalungu district since 1989.^23^ In comparison, Wakiso district borders the capital, and there is no previous link with the MRC.

Most other studies are among women and girls who could provide their own consent,^8^ whereas almost all our participants (98.1%) required parental consent due to their age. The proportion of parents who declined consent in our study is relatively high (43.1%), but among those whose parents did provide consent, the proportion of students providing assent was 79.4%. This is higher than the proportion of participants who used the cup in a study in Zimbabwe (7.2 %), similar to the objective estimated use of the cup in Kenya (70.9%) and lower than the reported usage in South African and Kenyan studies (>80%). This is likely because participants in the Zimbabwean study had to choose either the cup or reusable pads,^6^ whereas in our study, they had the option of receiving both products, and in both the South African and Kenyan studies, they were only provided with the cup.^7 12^

We found no evidence of a relationship of age with cup decline for parents and students, in contrast with the study in Zimbabwe which found that menstrual cup uptake was higher among 20–24 year-olds than among 16–19 year olds (16.6% vs 7.2%; p<0.001).^6^ The difference in the findings could be due to different study populations and settings coupled with sociocultural differences. Further, our participants were all in S2 at baseline, so older participants were likely to have had challenges with education and are not representative of other girls of their age.

The higher proportion of day students who declined the cup compared with boarding students could be due to external influences such as community members and/or parents who have reservations about insertion of the cup.^6 7 24^ Willingness to try the cup among boarding students may also be higher due to stronger peer influence in the boarding system which influences menstrual product use.^8^ The greater decline for the cup among students with fewer unmet menstrual needs measured by the MPNS score and better academic performance may be because these students have more menstrual product choices or are comfortable with their current choice. Further work on the associations between product use and dimensions of MH are needed, to assess how generalisable these findings are. Conversely, students with more positive attitudes towards MH were less likely to decline the cup. This may be due to better knowledge about menstruation and menstrual product choice, as well as the positive sociocultural norms.^6^ This means that efforts to increase awareness about MH and menstrual products would help to improve uptake of a menstrual cup.

We found no evidence of an association of decline for the cup with the SAMNS score on menstrual self-efficacy. This may be because the SAMNS is measuring girls’ confidence with the full menstrual experience and not only the one related with the hygiene management aspect.^17^ It is possible that school-level WASH facilities may be associated with declining a cup due to concerns about access to cleaning and washing space, but we found no evidence of this.

The strengths of this research are the large study size, high response rate, and inclusion of both parental consent and student assent. A limitation of this paper includes the exploration of parental consent and student assent/consent to menstrual cup uptake and not whether the student proceeded to use the menstrual cup after she assented, and if so for how long. This information is available for students in the intervention arm during trial follow-up and will be assessed separately using mixed-methods process evaluation data. In addition, we do not have data on the reason why parents or students declined to receive the menstrual cup. As this research was only conducted within two districts in Uganda whereby ethnicity is indicated to play a role in differentiating parental consent, these results may not be generalisable to other settings whereby the sociocultural environment may be very different.

## Conclusion

Our research highlights the importance of understanding contextual factors which are likely to affect parental and student consent/assent when planning menstrual cup interventions in school settings. Such factors are likely to include sociocultural norms, religion and existing MH practices and attitudes among students. To mitigate barriers to uptake, implementors should consider ways to meaningfully engage parents and community members in MH education around product choice, including the menstrual cup if this is being offered. The findings highlight the need for a choice of menstrual products when implementing MH interventions in schools as menstrual cups will not be the preferred menstrual product choice for all. Research is needed to understand reasons for uptake of MH product choices in different contexts and the cost and cost-effectiveness of school-based MH interventions that offer a choice of MH products.

## supplementary material

10.1136/bmjopen-2024-087438 online supplemental file 1

10.1136/bmjopen-2024-087438 online supplemental file 2

## Data Availability

Data will be available from the LSHTM Data Repository (https://datacompass.lshtm.ac.uk/) 12 months after trial completion for which the baseline data is used for this paper. Trial materials are available at the trial website (https://www.lshtm.ac.uk/research/centres-projects-groups/meniscus).
