# Supplementary material for: Factors associated with declining a menstrual cup among female students and their parents in Ugandan secondary schools: a cross-sectional study
Source: BMJ Open. 2024 Dec 5;14(12):e087438. doi: 10.1136/bmjopen-2024-087438 (PMC11624753; doi:10.1136/bmjopen-2024-087438)
Supplement: online supplemental file 1 [file bmjopen-14-12-s001.docx]

**Supplemental Figure 1. Number of post-menarchal female students recruited and analysed**

**Students with parental consent for the trial: N=3635**

797 Kalungu

2838 Wakiso

**Students aged 18+** (parental consent not applicable) consenting to trial: N=**70**

25 Kalungu

45 Wakiso

**Parental outcome:** Declined cup

**(N=1566)**

64 Kalungu

1502 Wakiso

**Parental outcome:** Consented to cup

**(N=2069)**

733 Kalungu

1336 Wakiso

**Student outcome:**

Assented to cup (**N=1,643**)

577 Kalungu

1,066 Wakiso

**Student outcome:**

Declined cup (**N=426**)

156 Kalungu

270 Wakiso

**Student outcome:**

Declined cup (**N=13**)

4 Kalungu

9 Wakiso

**Student outcome:**

Consented to cup (**N=57**)

21 Kalungu

36 Wakiso
