# Supplementary material for: Factors associated with declining a menstrual cup among female students and their parents in Ugandan secondary schools: a cross-sectional study
Source: BMJ Open. 2024 Dec 5;14(12):e087438. doi: 10.1136/bmjopen-2024-087438 (PMC11624753; doi:10.1136/bmjopen-2024-087438)
Supplement: online supplemental file 2 [file bmjopen-14-12-s002.docx]

**Supplemental Table 1. Factors associated with student menstrual cup decline.**

|  | **Total**  **N (%)** | **Declined cup**  **N (%)** | **Unadjusted**  **OR (95% CI)** | ***LRT***  ***p*-value** | **Adjusted^2^**  **OR (95% CI)** | ***LRT***  ***p*-value** |
| --- | --- | --- | --- | --- | --- | --- |
| N | 2,139 | 439 (20.5) |  |  |  |  |
| **Level 1:**  **Childhood and distal socio-demographic factors** | | | | | | |
| **District** |  |  |  |  |  |  |
| Kalungu | 758 (35.4) | 160 (21.1) | 1.00 |  |  |  |
| Wakiso | 1,381 (64.6) | 279 (20.0) | 0.99 (0.55, 1.79) | 0.97 |  |  |
| **Religion** |  |  |  |  |  |  |
| Catholic | 719 (33.6) | 137 (19.1) | 1.00 |  |  |  |
| Protestant/Born Again/SDA | 807 (37.7) | 170 (21.1) | 1.01 (0.76, 1.34) | 0.84 |  |  |
| Muslim | 604 (28.2) | 132 (21.9) | 1.09 (0.80, 1.50) |  |  |  |
| None/Other | 9 (0.4) | 0 (0.0) | … |  |  |  |
| **Ethnicity** |  |  |  |  |  |  |
| Muganda | 1,436 (67.1) | 299 (20.8) | 1.00 |  |  |  |
| Non Muganda | 703 (32.9) | 140 (19.9) | 0.93 (0.73, 1.19) | 0.58 |  |  |
| **Caregiver education** | |  |  |  |  |  |
| Primary or less | 525 (24.5) | 98 (18.7) | 1.00 |  | 1.00 |  |
| Secondary | 704 (32.9) | 127 (18.0) | 1.01 (0.74, 1.38) | 0.02 | 1.04 (0.76, 1.42) | 0.02 |
| More than secondary | 552 (25.8) | 122 (22.1) | 1.24 (0.89, 1.72) |  | 1.29 (0.93, 1.79) |  |
| Don't know | 358 (16.7) | 92 (25.7) | 1.62 (1.14, 2.30) |  | 1.65 (1.16, 2.35) |  |
| **Social Economic Status (SES)** |  |  |  |  |  |  |
| Lowest | 436 (20.4) | 99 (22.7) | 1.00 |  |  |  |
| Medium-low | 471 (22.0) | 85 (18.1) | 0.74 (0.52, 1.04) | 0.53 |  |  |
| Medium | 418 (19.5) | 83 (19.9) | 0.82 (0.58, 1.18) |  |  |  |
| Medium-high | 414 (19.4) | 90 (21.7) | 0.87 (0.61, 1.24) |  |  |  |
| Highest | 400 (18.7) | 82 (20.5) | 0.83 (0.57, 1.21) |  |  |  |
| **Type of caregiver** |  |  |  |  |  |  |
| Mother | 1,251 (58.5) | 250 (20.0) | 1.00 |  |  |  |
| Other^1^ | 888 (41.5) | 189 (21.3) | 1.11 (0.89, 1.40) | 0.35 |  |  |
| **Age categories (years)** |  |  |  |  |  |  |
| ≤15 | 1,026 (48.0) | 204 (19.9) | 1.00 |  |  |  |
| >15 | 1,113 (52.0) | 235 (21.1) | 1.14 (0.91, 1.44) | 0.26 |  |  |
| **Household size** |  |  |  |  |  |  |
| <5 people | 641 (30.0) | 139 (21.7) | 1.00 |  |  |  |
| 6-10 people | 1,296 (60.6) | 257 (19.8) | 0.85 (0.66, 1.09) | 0.43 |  |  |
| 10+ people | 202 (9.4) | 43 (21.3) | 0.92 (0.61, 1.39) |  |  |  |
| **Number of meals eaten on the previous day** |  |  |  |  |  |  |
| One or fewer | 386 (18.1) | 65 (16.8) | 1.00 |  |  |  |
| Two | 1,077 (50.4) | 224 (20.8) | 1.37 (0.99, 1.89) | 0.09 |  |  |
| Three or more | 676 (31.6) | 150 (22.2) | 1.43 (1.01, 2.01) |  |  |  |
| **Level 1: Distal school level factors** | | | | | | |
| **School ownership** |  |  |  |  |  |  |
| Government | 780 (36.5) | 149 (19.1) | 1.00 |  |  |  |
| Private | 1,359 (63.5) | 290 (21.3) | 0.92 (0.52, 1.63) | 0.78 |  |  |
| **Water in toilet block** | | |  |  |  |  |
| No | 1,512 (70.7) | 302 (20.3) | 1.00 |  |  |  |
| Yes(piped/container) | 627 (29.3) | 137 (21.1) | 1.40 (0.90, 2.18) | 0.13 |  |  |
| **Separate toilet blocks for girls and boys** | | |  |  |  |  |
| No | 156 (7.3) | 45 (28.9) | 1.00 |  |  |  |
| Yes | 1,983 (92.7) | 394 (19.9) | 0.57 (0.24, 1.35) | 0.21 |  |  |
| **Private menstrual changing space available** | | |  |  |  |  |
| No | 1,746 (81.6) | 343 (19.6) | 1.00 |  |  |  |
| Yes | 393 (18.4) | 96 (24.4) | 1.65 (0.84, 3.22) | 0.14 |  |  |
| **Schooling category** | |  |  |  |  |  |
| Boarding | 957 (44.7) | 193 (20.2) | 1.00 |  | 1.00 |  |
| Day | 1,182 (55.3) | 246 (20.8) | 1.38 (1.04, 1.82) | 0.03 | 1.40 (1.07, 1.84) | 0.01 |
| **School-level UNEB assessment score** | | | |  |  |  |
| Low (≤-3) | 1,334 (62.4) | 260 (19.5) | 1.00 |  |  |  |
| High (>-3) | 805 (37.6) | 179 (22.2) | 1.09 (0.61, 1.96) | 0.77 |  |  |
| **Individual-level UNEB assessment score** | | | |  |  |  |
| Low (≤-3) | 1,069 (50.0) | 196 (18.3) | 1.00 |  | 1.00 |  |
| High (>-3) | 1,070 (50.0) | 236 (22.7) | 1.29 (1.01, 1.64) | 0.04 | 1.29 (1.01, 1.65) | 0.04 |
| **Level 2: Proximal menstrual related factors** | | | | | | |
| **Knowledge questions answered correctly (out of 9)** | | | |  |  |  |
| <7 | 1,795 (83.9) | 372 (20.7) | 1.00 |  |  |  |
| ≥7 | 344 (16.1) | 67 (19.5) | 0.90 (0.66, 1.23) | 0.51 |  |  |
| **Attitude questions answered positively (out of 3)** | | | |  |  |  |
| ≥2 | 1,070 (50.0) | 196 (18.3) | 1.00 |  | 1.00 |  |
| <2 | 1,069 (50.0) | 243 (22.7) | 1.35 (1.08, 1.70) | 0.01 | 1.46 (1.16, 1.83) | 0.001 |
| **Age at menarche** | | | |  |  |  |
| ≤12 | 435 (20.3) | 74 (17.0) | 1.00 |  |  |  |
| 13 | 760 (35.5) | 168 (22.1) | 1.36 (0.99, 1.87) | 0.27 |  |  |
| 14 | 702 (32.8) | 148 (21.1) | 1.30 (0.94, 1.80) |  |  |  |
| 15+ | 242 (11.3) | 49 (20.3) | 1.22 (0.79, 1.87) |  |  |  |
| **Knew about menstruation before first period** | | | |  |  |  |
| No | 604 (28.2) | 134 (22.2) | 1.00 |  |  |  |
| Yes | 1,535 (71.8) | 305 (20.0) | 0.85 (0.66, 1.08) | 0.19 |  |  |
| **Used a menstrual cup at LMP** | | |  |  |  |  |
| No | 2,126 (99.4) | 438 (20.6) | 1.00 |  |  |  |
| Yes | 13 (0.6) | 1 (7.7) | 0.40 (0.05, 3.22) | 0.33 |  |  |
| **Used disposable products at LMP** | | |  |  |  |  |
| No | 362 (16.9) | 66 (18.2) | 1.00 |  |  |  |
| Yes | 1,777 (83.1) | 373 (21.0) | 1.14 (0.83, 1.55) | 0.42 |  |  |
| **Used reusable products (including a menstrual cup) at LMP^3^** | | | |  |  |  |
| No | 1,428 (66.8) | 311 (21.8) | 1.00 |  |  |  |
| Yes | 711 (33.2) | 128 (18.0) | 0.79 (0.62, 1.01) | 0.06 |  |  |
| **MPNS score** |  |  |  |  |  |  |
| Low (≤2.1) | 1,147 (53.6) | 211 (18.4) | 1.00 |  | 1.00 |  |
| High (>2.1) | 992 (46.4) | 228 (23.0) | 1.31 (1.04, 1.64) | 0.02 | 1.36 (1.08, 1.72) | 0.01 |
| **SAMNS-26 score** |  |  |  |  |  |  |
| Low (≤6.2) | 1,115 (52.1) | 228 (20.5) | 1.00 |  |  |  |
| High (>6.2) | 1,024 (47.9) | 211 (20.6) | 0.98 (0.78, 1.22) | 0.84 |  |  |
| **SDQ score** |  |  |  |  |  |  |
| Low (≤12) | 1,198 (56.0) | 261 (21.8) | 1.00 |  |  |  |
| High (>12) | 941 (44.0) | 178 (18.9) | 0.79 (0.63, 0.99) | 0.04 |  |  |

*^1^Includes father, sibling, grand parent, aunt, uncle, and self*

*^2^Variables are adjusted for those at the same or lower levels (e.g., variables at Level 2 are adjusted for each other and for those at Level 1 that were retained in Level 1 final model).*

*^3^Thirteen (0.6%) students reported to have used a menstrual cup at LMP, and 350/2139 (16.4%) reported using both disposable and reusable products at LMP*
