# Supplementary material for: Factors associated with declining a menstrual cup among female students and their parents in Ugandan secondary schools: a cross-sectional study
Source: BMJ Open. 2024 Dec 5;14(12):e087438. doi: 10.1136/bmjopen-2024-087438 (PMC11624753; doi:10.1136/bmjopen-2024-087438)
Supplement: Supplementary file 3 [file bmjopen-2024-087438.reviewer_comments.pdf]

PEER REVIEW HISTORY

BMJ Open publishes all reviews undertaken for accepted manuscripts. Reviewers are asked to complete a checklist review form and are provided with free text boxes to elaborate on their assessment. These free text comments are reproduced below.

ARTICLE DETAILS

Title (Provisional)

Factors associated with declining a menstrual cup among female students and their parents in Ugandan secondary schools: A cross-sectional study

Authors

Mugenyi, Levicatus; Tembo, Mandikudza; Nelson, Kate; Thomas, Katherine; Kansiime, Catherine; Lagony, Stephen; Mpaata, Alex Muleyi; Belfield, Sophie; Nakalema, Shamirah; Akech, Agnes; Torondel, Belen; Weiss, Helen Anne

---

VERSION 1 - REVIEW

**Reviewer**	**1**
**Name**	**Bannow, Bethany Samuelson**
**Affiliation**	**Oregon Health & Science University**
**Date**	**20-Jul-2024**
**COI**	**NA**

Mugenyi et al report on responsiveness of 3705 female students (or parents) in Uganda to offers of free menstrual cups. They found that 20.5% of students declined the cup and that this varied by district and ethnicity.

The authors are to be congratulated on this important work to better understand knowledge, beliefs and acceptability of a menstrual cup. The study is well-designed, the writing is clear and concise and the work is important. However, I'm confused as to why the main focus of this paper is acceptability of a menstrual cup, which, while interesting and potentially having a public health impact in areas where access to other products is restricted, seems far less significant to this reviewer than the rich data in table 2, which identify many important factors for educational and resource interventions. Furthermore, discussion on the MPNS, SDQ, and SAMNS-26 scores, putting these results in context with those of other populations and identifying key areas for intervention, is lacking and would do much to improve the paper. After reading the abstract I was under the impression that this paper would be better suited as a letter as it implies looking at a single outcome with a few different predictors. However, in a way, this is a cross-sectional study of menstrual norms, practices, and access, which is ultimately far more interesting and important

(although certainly discussion of those who declined participation is important as well). This reviewer recommends reframing the paper as a discussion of all these findings, rather than primarily being about the menstrual cup acceptability.

Reviewer 2

Name Jahan , Farjana

Affiliation International Centre for Diarrhoeal Disease Research Bangladesh, Environmental Intervention Unit, Infectious Disease Division

Date 08-Aug-2024

COI NA

Overall Comments:

Menstrual cup as a menstrual hygiene management material is comparatively new introduction to LMICs, so I congratulate the authors for conducting this unique study. The introduction provides clear insights about the context, however the method section is not clear enough to understand the design of the study. The results could have considered the other essential factor influencing the uptake. To improve clarity and structure, it is advisable to make major revisions, particularly in further emphasizing on the analysis and improving the discussion section.

Specific comments:

• Title- the title does not reflect the overall study, the authors did not look at the uptake, as it did not look at the usage, they might agreed but not used it later. I believe it's their willingness to receive and use menstrual cup, I suggest to revisit the title.

• Introduction - Rephrase the sentences in Lines 81-88 to enhance their clarity. Readability could be improved by simplifying the existing structure. Revise Lines 90-101 to offer a more concise summary of the factors that influence the use of menstrual cups in low and middle-income countries (LMICs). The information is valuable; however, it could be simplified to facilitate comprehension.

• Methods- Please clarify what do you mean by minimal WASH facilities, are you following JMP criteria for determining the minimal WASH facility? Were the baseline survey conducted among the parents who consented, if that is the case how did you evaluate the factors among who did not consent, the method section is not clear at all. The scoring method of different scales that are used in this should be elaborated.

• Results- Usage of menstrual cup can be strongly associated with the access to cleaning and washing space at school also at home. If the school does not have any washing space it is unlikely they will change the cup at school.

What is the rational for using a cut off of <7 and >7 for knowledge questions? Same question applies for attitude questions. How did the team decide that cut off, does it mean those answered <7 correct answers have low knowledge?

The percentage of users using disposable (88.1%) and reusable products (33%) at LMP is confusing, does it mean there is a percentage of students who used both disposable and reusable at LMP? The authors should provide that percentage.

• Chronology of discussion section should be improved, strength and limitation can be discussed at the end of the discussion, currently it's not synchronized.

• Add a Separate Conclusion Section: Explain the implications for future interventions and research in a separate conclusion section. Include Lines 366-374's recommendations and future directions. This will assist readers in understanding the study's practical applications and next steps.

• Overall language and grammar of the manuscript should be improved.

VERSION 1 - AUTHOR RESPONSE

We thank the reviewers for their helpful and constructive comments. We have revised the manuscript, which is substantially improved by incorporating the changes recommended by the reviewers. Below, we provide a point-by-point response to the reviewers' comments and reference corresponding line numbers in the text of the manuscript with these changes.

Reviewer: 1
Dr. Bethany Samuelson Bannow, Oregon Health & Science University
Comments to the Author:
Mugenyi et al report on responsiveness of 3705 female students (or parents) in Uganda to offers of free menstrual cups. They found that 20.5% of students declined the cup and that this varied by district and ethnicity.
The authors are to be congratulated on this important work to better understand knowledge, beliefs and acceptability of a menstrual cup. The study is well-designed, the writing is clear and concise and the work is important.
Response: Thank you for these comments

However, I'm confused as to why the main focus of this paper is acceptability of a menstrual cup, which, while interesting and potentially having a public health impact in areas where access to other products is restricted, seems far less significant to this reviewer than the rich data in table 2, which identify many important factors for educational and resource interventions.
Response: We thank the reviewer for noting that the study contains a wealth of data on menstrual health, and we will be reporting in detail on menstrual health in this population, including the impact of the intervention on menstrual health, in subsequent papers. We have clarified this in the paper (Lines 112-113) and also expanded our description of the intervention to clarify that this was a comprehensive menstrual health intervention of which the offer of a menstrual cup was just one component (Lines 119-127). As the reviewer comments, acceptability of the menstrual cup is an interesting and important topic in itself, and the trial data provides a unique opportunity to look at characteristics of participants who agreed to take part

in the trial, but declined receiving a cup. We have explained the rationale for focusing on acceptability of the cup more clearly in the background (Lines 108-113).

Furthermore, discussion on the MPNS, SDQ, and SAMNS-26 scores, putting these results in context with those of other populations and identifying key areas for intervention, is lacking and would do much to improve the paper.
Response: We agree that the use of these scales is a key feature of our trial data, and the impact of the intervention on these outcomes and further description of the properties of these scales are the topic of separate papers. This paper is the first to our knowledge to assess the association of the MPNS, SAMNS-26 and SDQ scores (as exposures) with acceptability of the menstrual cup. We have expanded the discussion on the associations between declining cup use with the MPNS and SAMNS, including the need for further work on the association of validated measures for menstrual health with cup acceptability (Lines 378-380).

After reading the abstract I was under the impression that this paper would be better suited as a letter as it implies looking at a single outcome with a few different predictors. However, in a way, this is a cross-sectional study of menstrual norms, practices, and access, which is ultimately far more interesting and important (although certainly discussion of those who declined participation is important as well).
Response: We thank the reviewer for appreciating the richness of the data. We chose to focus this paper on menstrual cup acceptability as the cup is being increasingly offered to girls and women, but there are few data on its acceptability and factors associated with this. We have explained this more clearly in the background (lines 110-113). Also, we have clarified that data were collected from all female students (and on their primary caregivers) that consented to the trial (some of whom did not consent to the cup), meaning data on factors among those who never consented to the cup were available and could be evaluated (lines 189-193).

This reviewer recommends reframing the paper as a discussion of all these findings, rather than primarily being about the menstrual cup acceptability.
Response: We thank the reviewer for this suggestion and have expanded the discussion to include a more detailed discussion of the association of menstrual health and menstrual cup decline (Lines 369-382) and the need for further work on this topic. Given the evidence gap on the acceptability of the menstrual cup, we prefer to retain our original objectives.

Reviewer: 2
Dr. Farjana Jahan, International Centre for Diarrhoeal Disease Research Bangladesh,
International Centre for Diarrhoeal Disease Research Bangladesh

Comments to the Author:
Overall Comments:
Menstrual cup as a menstrual hygiene management material is comparatively new introduction to LMICs, so I congratulate the authors for conducting this unique study. The introduction provides clear insights about the context, however the method section is not clear enough to understand the design of the study. The results could have considered the other essential factor influencing the uptake. To improve clarity and structure, it is advisable to make major revisions, particularly in further emphasizing on the analysis and improving the discussion section.
Response: Thank you for your comments. We have expanded the methods section by adding in the following 1) a more detailed explanation of the use of the baseline data to assess factors associated with declining cup consent and that the cup was just one component of the intervention (lines 116-127); 2) That consent to the cup was optional among those who consented to the other trial procedures (lines 165-169)); 3) More details on the menstrual health

tools used (lines 183-188); and 4) rewording of the statistical analysis section to clarify the model building strategy (lines 240-247).

We have revised the discussion throughout, including a more detailed summary paragraph including discussion of the effect-modification by ethnicity (lines 326-340), revised discussion comparing our findings with those of other studies and reasons for differences (lines 353-361) the finding with age (lines 362-367) and the associations with menstrual health (lines 368-383).

Specific comments:
• Title- the title does not reflect the overall study, the authors did not look at the uptake, as it did not look at the usage, they might agreed but not used it later. I believe it's their willingness to receive and use menstrual cup, I suggest to revisit the title.
Response: We agree with the reviewer, and have revised the title as follows "Factors associated with declining a menstrual cup among female students and their parents in Ugandan secondary schools: A cross-sectional study". We have also clarified in the paper that we only look at willingness to receive the cup, not reported usage (lines 368-369).

• Introduction - Rephrase the sentences in Lines 81-88 to enhance their clarity. Readability could be improved by simplifying the existing structure. Revise Lines 90-101 to offer a more concise summary of the factors that influence the use of menstrual cups in low and middle-income countries (LMICs). The information is valuable; however, it could be simplified to facilitate comprehension.
Response: We thank the reviewer for raising this and have improved the readability here and throughout the paper. Specifically, we have revised the description of cup use in LMICs on lines 89-103.

• Methods- Please clarify what do you mean by minimal WASH facilities, are you following JMP criteria for determining the minimal WASH facility?
Response: By minimal WASH facility we mean having an improved water source and sex-specific sanitation facilities that are functional, usable, and accessible to female students. We have included this on lines 139-141).

Were the baseline survey conducted among the parents who consented, if that is the case how did you evaluate the factors among who did not consent, the method section is not clear at all.
Response: Data were collected from all female students with parental consent and assent (or student consent if aged >=18 years) for the main trial. Some of these did not have consent/assent for the cup, as this had a separate opt-in consent/assent procedure. We therefore have data from the survey for participants both with and without consent for the cup. We have clarified this in the methods (lines 165-169 and 189-193).

The scoring method of different scales that are used in this should be elaborated.
Response: We have explained the scales on lines 184-188, and their scoring on lines 211-227.

• Results- Usage of menstrual cup can be strongly associated with the access to cleaning and washing space at school also at home. If the school does not have any washing space it is unlikely they will change the cup at school.
Response: We agree with the reviewer that access to cleaning and washing space at school and at home can be associated with use of menstrual cup. Whilst we did not collect data on home-level WASH facilities, we did collect school-level data on WASH facilities such as availability of water in a toilet block. As shown in Table 3, there was no evidence of an association with having

water available in the toilet block, or separate toilets for girls and boys. We have added this into the discussion (lines 380-382).

What is the rational for using a cut off of <7 and >7 for knowledge questions? Same question applies for attitude questions. How did the team decide that cut off, does it mean those answered <7 correct answers have low knowledge?
Response: As few girls answered all 8-9 knowledge questions correctly (3.6%), we chose a cutoff of >7 to improve the statistical power to detect an association with the outcome, whilst still reflecting "good" knowledge.

The percentage of users using disposable (88.1%) and reusable products (33%) at LMP is confusing, does it mean there is a percentage of students who used both disposable and reusable at LMP? The authors should provide that percentage.
Response: Yes, 350/2139 (16.4%) used both disposable and reusable products at LMP. This result has been added as a footnote under Table 3 where we report statistics using disposable (83.1% not 88.1% as quoted by the reviewer) and reusable (33.2%).

• Chronology of discussion section should be improved, strength and limitation can be discussed at the end of the discussion, currently it's not synchronized.
Response: We thank the reviewer for this comment and have revised the discussion throughout, including moving the strengths and limitations to a paragraph at the end of the discussion (lines 383-391).

• Add a Separate Conclusion Section: Explain the implications for future interventions and research in a separate conclusion section. Include Lines 366-374's recommendations and future directions. This will assist readers in understanding the study's practical applications and next steps.
Response: We provide a separate conclusion section on lines 392-402 which includes implications for future interventions and research.

• Overall language and grammar of the manuscript should be improved.
Response: We have revised the language and grammar throughout the manuscript.

VERSION 2 - REVIEW

**Reviewer**	**1**
**Name**	**Bannow, Bethany Samuelson**
**Affiliation**	**Oregon Health & Science University**
**Date**	**05-Nov-2024**
**COI**	

The paper is improved by the changes made.
